# Differentiation and transdifferentiation potentials of cancer stem cells

**DOI:** 10.18632/oncotarget.6098

**Published:** 2015-10-12

**Authors:** Zhengjie Huang, Tiantian Wu, Allan Yi Liu, Gaoliang Ouyang

**Affiliations:** ^1^ Department of Surgical Oncology, First Affiliated Hospital of Xiamen University, Xiamen, China; ^2^ State Key Laboratory of Cellular Stress Biology, Innovation Center for Cell Signaling Network, School of Life Sciences, Xiamen University, Xiamen, China

**Keywords:** cancer stem cell, differentiation, transdifferentiation, stromal cells, tumor microenvironment

## Abstract

Tumor cells actively contribute to constructing their own microenvironment during tumorigenesis and tumor progression. The tumor microenvironment contains multiple types of stromal cells that work together with the extracellular matrix and local and systemic factors to coordinately contribute to tumor initiation and progression. Tumor cells and their stromal compartments acquire many genetic and/or epigenetic alternations to facilitate tumor growth and metastasis. The cancer stem cell (CSC) concept has been widely applied to interpreting tumor initiation, growth, metastasis, dormancy and relapse. CSCs have differentiation abilities to generate the original lineage cells that are similar to their normal stem cell counterparts. Interestingly, recent evidence demonstrates that CSCs also have the potential to transdifferentiate into vascular endothelial cells and pericytes, indicating that CSCs can transdifferentiate into other lineage cells for promoting tumor growth and metastasis in some tissue contexts instead of only recruiting stromal cells from local or distant tissues. Although the transdifferentiation of CSCs into tumor stromal cells provides a new dimension that explains tumor heterogeneity, many aspects of CSC transdifferentiation remain elusive. In this review, we summarize the multi-lineage differentiation and transdifferentiation potentials of CSCs as well as discuss their potential contributions to tumor heterogeneity and tumor microenvironment in tumor progression.

## INTRODUCTION

Cancer stem cells (CSCs), also known as tumor-initiating cells or tumor-propagating cells, refer to a subpopulation of tumor cells that have abilities to self-renew, differentiate and seed new tumors. Accumulating evidence demonstrates that CSCs contribute to tumorigenesis, metastasis, dormancy and relapse [1–3]. CSCs were first identified in acute myeloid lymphoma [4, 5] and were then isolated in a variety of solid tumors including breast [6], brain [7, 8], colon [9, 10], liver cancers [11, 12], melanoma [13] and some other tumors.

Because CSCs exhibit self-renewal and multi-lineage differentiation abilities that are similar to their normal stem cell counterparts, CSCs were initially thought to originate from normal stem cells. Transducing MLL-AF9 fusion protein into myeloid progenitors gives rise to leukemia *in vivo*, indicating that progenitors can convert to leukemia stem cells [14]. Huntly *et al.,* reported that MOZ-TIF2, but not BCR-ABL, transforms myeloid progenitors into leukemia initiating cells [15]. All of these studies in mouse models suggest that progenitor cells contribute to the CSC pool by genetic and/or epigenetic hits. However, CSCs do not definitely originate from normal stem cells or progenitors. Mani *et al*., discovered that the ectopic expression of transcription factors, such as Twist1 and Snail, or treatment with TGF-β in mammary epithelial cells or cancer cells can induce stem cell-like or cancer stem cell-like phenotypes [16]. Terminally differentiated cortical astrocytes and neurons that are transduced by oncogenes give rise to glioblastoma (GBM) in mouse models, indicating that these cells may dedifferentiate into CSCs [17]. There was another report that intestinal epithelial cells covert to stemness state and drive tumorigenesis through oncogenic hits, such as Ras or NF-κB activation [18]. Skin fibroblasts that stably express hTERT, H-RasV12 and SV40 LT and ST antigens *in vitro* acquire CSC properties, undergoing multi-lineage differentiation and generating hierarchically organized tumors *in vivo* [19]. Thus, the acquisition and accumulation of genetic and/or epigenetic alterations can covert cancer cells, even some normal cells, to a stemness state by dedifferentiation, indicating that this dedifferentiation program can generate CSCs. In addition, cell fusion is a common event in mammals; therefore, CSCs may originate from the fusion between normal stem cells and somatic cells. However, it remains unclear whether this fusion actually contributes to the CSC pool because tracing cell fusion *in vivo* still involves many obstacles. Therefore, CSCs may originate from their normal stem cells, progenitors and/or differentiated somatic cells.

**Figure 1:**
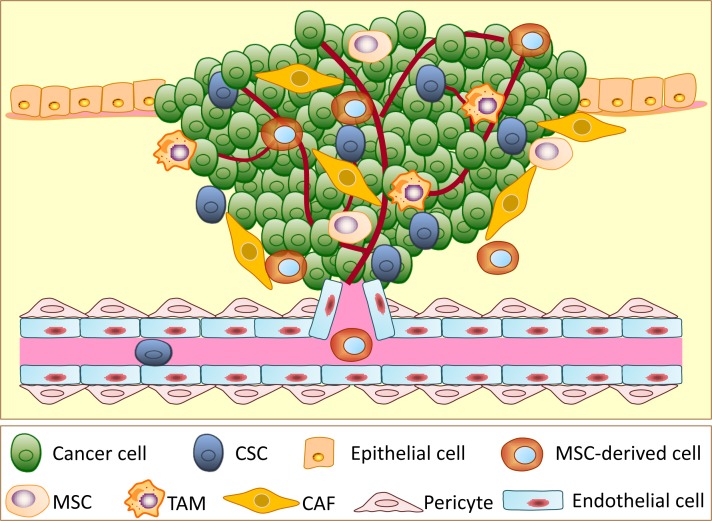
A schematic illustration showing the different types of cells involved in tumor progression Tumors are very complicated neoplasms that not only consist of cancer stem cells (CSCs) and non-stem cancer cells, they also have numerous types of stromal cells, including cancer-associated fibroblasts (CAFs), endothelial cells, pericytes, tumor-associated macrophages (TAMs), mesenchymal stem cells (MSCs), MSC-derived cells and other stromal cells.

**Figure 2:**
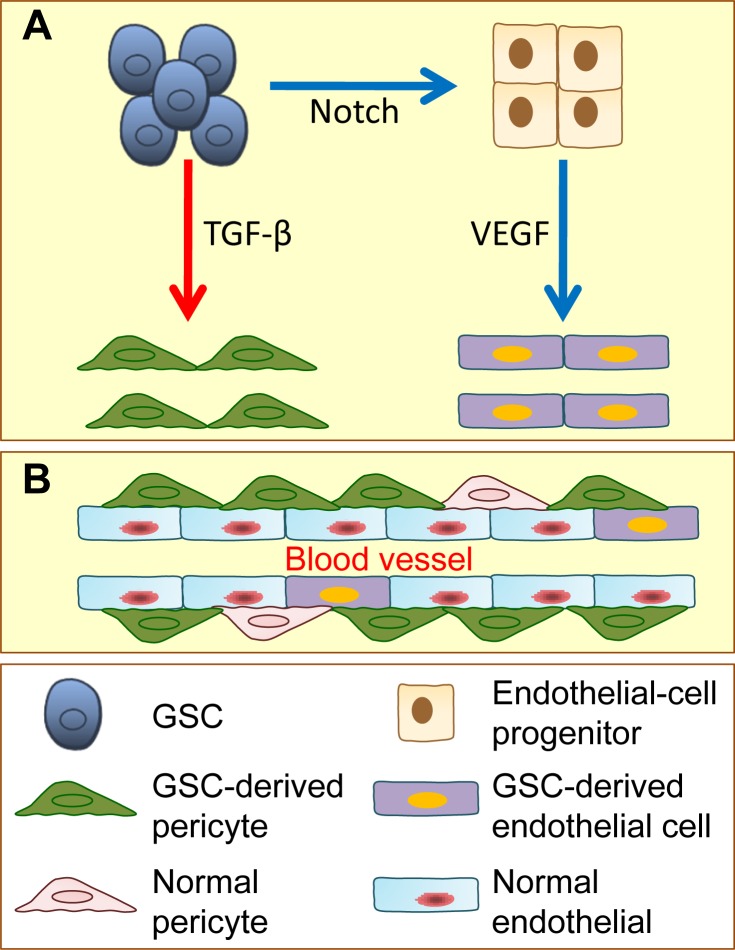
Glioblastoma stem cells (GSCs) have the potential to give rise to endothelial cells and pericytes **A.** With the induction of activated Notch signaling, GSCs transdifferentiate into endothelial-cell progenitors, which further differentiate into endothelial cells by VEGF induction. GSCs also have potential to generate pericytes. When induced by TGF-β, GSCs, which are recruited by endothelial cells via SDF-1/CXCR4 chemokine signaling, can transdifferentiate into pericytes. **B.** GSC-derived pericytes and GSC-derived endothelial cells, together with normal pericytes and endothelial cells, contribute to tumor vessel function and tumor development.

Tumors are not regarded as a mere collection of homogenous cancer cells. Increasing evidence supports that the tumor contains heterogeneous cancer cells and different types of stromal cells (Figure 1) [20, 21]. Cancer cells recruit stromal cells from bone marrow or surrounding tissues to construct their own microenvironment and coordinately contribute to tumor initiation and progression. In addition to recruiting stromal cells to the microenvironment, cancer cells can fuse with or transdifferentiate into several types of stromal cells and gain partial properties of these stromal cells to favor cancer cell survival, proliferation, invasion and metastasis. Accumulating evidence has revealed that CSCs have a multi-lineage differentiation ability that is similar to normal stem cells. Moreover, CSCs have potential to transdifferentiate into vascular endothelial cells and pericytes *in vitro* and *in vivo* (Figure 2) [22–26]. Furthermore, various differentiated cells have been directly reprogrammed from one cell type into another with the induction of potent transcription factors [27]. Therefore, CSC theory provides new insight into the tumor heterogeneity because of the multi-lineage differentiation and transdifferentiation potentials of CSCs. Here, we enumerate known evidence for the differentiation or transdifferentiation of CSCs in tumors and discuss the potential contributions of CSC differentiation and transdifferentiation in the tumor heterogeneity as well as the microenvironment in tumor progression.

## DIFFERENTIATION POTENTIALS OF CANCER STEM CELLS

According to the CSC theory, CSCs can differentiate into cancer cells and are responsible for tumor growth and metastasis. Dick and colleagues identified a CD34^+^/CD38^−^ subpopulation from patient samples as acute myeloid leukemia stem cells. This subpopulation can differentiate into leukemia cells *in vivo* [5]. Leukemia stem cells are also well investigated in chronic myeloid leukemia (CML). In CML patients, granulocyte-macrophage progenitors serve as leukemia stem cell candidates and differentiate and promote progression of CML into blast crisis stage [28]. The differentiation of CSCs into cancer cells has also been detected in numerous solid tumors. Al-Hajj *et al*., discovered that CD44^+^/CD24^− or low^/Lin^−^ cells differentiate into non-tumorigenic cancer cells [6]. In brain tumors, CD133^+^ tumor initiating cells give rise to multi-lineage CD133^−^ cells (astrocytes, oligodendrocytes or neurons) *in vitro* and *in vivo* [8, 29]. In colon cancer, the CD44^+^/EpCAM^+^ or CD133^+^ subpopulation initiates tumorigenesis and differentiates into colon cancer cells [9, 10].

The differentiation of CSCs into cancer cells has been validated in other types of cancers, including pancreatic [30], prostate [31], lung [32] and liver cancers [11, 12]. For example, single-cell-cloned liver CSCs possess colony-forming ability *in vitro* and serial transplantation ability *in vivo*. When treat with the different tumor cell/tissue-derived conditioned medium, the single-cell-cloned CSCs are able to differentiate into respective type of tumor cells [33]. In addition to in carcinoma, the differentiation of CSCs has also been reported in sarcoma. Sarcoma originates from transformed mesenchymal cells that display multi-lineage differentiation abilities that are similar to mesenchymal stem cells (MSCs). In Ewing's sarcoma, CD133^+^ subpopulation establishes tumors *in vivo* with hierarchy cell organization and exhibits MSC plasticity to undergo adipogenic, osteogenic and chondrogenic differentiation *in vitro* [34]. Naka *et al*., discovered that Yamoto-synovial sarcoma cells are enriched for CSCs and efficiently drive tumor formation *in vivo*. Silencing SS18-SSX genes in synovial sarcoma stem cells induces multiple-lineage differentiation *in vitro* that is similar to MSCs [35]. Collectively, CSCs can differentiate into cancer cells *in vitro* and initiate tumorigenesis with the hierarchy organization of cancer cells *in vivo*.

CSCs share many properties with normal stem cells, including persistent activation of Notch, Hedgehog, and Wnt pathways, which are involved in the regulation of differentiation and self-renewal of stem cells [36]. Fibroblast-derived Periostin promotes self-renewal of breast CSCs and the lung metastasis of mouse breast tumors by augmenting Wnt signaling [37]. Similar to normal stem cells, CSCs locate at functional niches that provide indispensable cues to regulate and maintain the cellular hierarchy. CSCs will leave off self-renewal and undergo lineage specification when they are away from their niches [38]. Cancer-associated fibroblasts (CAFs) constitute a supporting niche for lung cancer stemness via paracrine signaling. Removal of CAFs results in the differentiation of lung CSCs [39]. Colon cancer cells adjoining stromal myofibroblasts show high activity of the Wnt pathway, indicating that extrinsic cues are involved in the regulating of Wnt activity and stemness of CSCs. Colon CSCs can be induced into more differentiated tumor cells by the microenvironment [40]. Lombardo et al., found that BMP4 promotes differentiation of human colorectal CSCs, similar to it does in normal colonic stem cells [41]. Interestingly, BMP promotes neural stem cell differentiation but is highly expressed in glioblastoma. A recent report demonstrates that Gremlin1, an antagonist of BMP, is specifically expressed by glioblastoma CSCs and counteracts the effects of BMP-induced differentiation, maintaining the dynamic balance between glioblastoma tumor proliferation and glioblastoma hierarchies [38]. MicroRNAs (miRNAs) also play important roles in the regulation of the stemness and differentiation of CSCs. These miRNAs can be divided into two groups: pluripotent miRNAs and pro-differentiation miRNAs. Pluripotent miRNAs suppress cell differentiation but promote proliferation and self-renewal of CSCs. Pro-differentiation miRNAs facilitate differentiation of CSCs [42]. These reports demonstrate that CSCs can differentiate into more differentiated tumor cells and that this process is regulated by the extrinsic and intrinsic cues.

## TRANSDIFFERENTIATION POTENTIALS OF CANCER STEM CELLS

### Endothelial cells

Solid tumors exhibit diverse patterns of neovascularization, including sprouting angiogenesis, vasculogenesis, intussusception, vessel co-option, vascular mimicry and CSC transdifferentiation [43]. Vascular mimicry is a process in which tumor cells incorporate into blood vessels to form a vascular structure that is similar to normal vessels [44, 45]. Vascular mimicry was first identified in melanoma wherein some melanoma cells co-express both tumor cell and endothelial cell markers. These melanoma cells can form vascular tube-like structures and promote tumor growth and metastasis [46]. Since then, vascular mimicry has been discovered in a variety of human tumors, including breast, ovarian and lung cancers, GBM and sarcomas, indicating that tumor cells may exhibit cell plasticity. Human neural stem cells have been shown to transdifferentiate into endothelial cells independent of the cell fusion mechanism [47]. As a result, it is reasonable to speculate that CSCs may directly contribute to the presence of endothelial cells in tumor vessels. Indeed, CD105^+^ renal CSCs can generate endothelial cells *in vitro* and give rise to vessels with a human origin *in vivo* [48]. Bussolati *et al*., further discovered that human breast CSCs undergo endothelial differentiation to generate functional endothelial cells *in vitro* and *in vivo* [49]. The authors employed mammosphere culture to enrich the breast CSCs and successfully obtained breast CSC-derived endothelial cells that express several endothelial markers (e.g., CD31, VE-Cadherin, CD105 and vWF). Importantly, breast CSCs give rise to endothelial cells in NOD/SCID mice. Moreover, ovarian CSCs also serve as vascular progenitor cells [50]. CD44^+^ ovarian CSCs can form CD34^+^ blood vessels in xenograft tumor models. CD44^+^ ovarian CSCs do not express any endothelial progenitor cell markers, but they can differentiate into endothelial cells in Matrigel. Furthermore, these CD44^+^ cells form vessel-like structures in a VEGF-independent manner. These three papers provide evidence for the endothelial differentiation of CSCs; however, they lack robust *in vivo* and clinical evidence that CSCs directly contribute to tumor angiogenesis via transdifferentiation. In 2010, studies on GBM further illustrated that GBM stem cells (GSCs) generate endothelial cells in tumor angiogenesis [22, 23]. Ricci-Vitiani *et al*., reported that a large proportion of the endothelial cells in GBM harbors the same chromosomal alterations as in tumor cells. They isolated CD133^+^/CD31^−^ cells from GBM samples that differentiate into functional endothelial cells *in vitro*. This transdifferentiation event was further validated *in vivo* using a xenogarft model in which human origin of endothelial cells was detected without cell fusion. In addition, selectively targeting GBM stem cell-derived endothelial cells dramatically impairs tumor growth [22]. Wang *et al*., [23] discovered that CD105^+^ endothelial cells in GBM samples have GBM genetic mutations, indicating that these endothelial cells are not derived from normal endothelial cells. The purified CD133^+^/CD144^−^ GBM cells can transdifferentiate into endothelial cells *in vitro* and *in vivo* via endothelial progenitor cell status without nuclear fusion. Activated Notch signaling contributes to the transdifferentiation of GSCs into endothelial progenitors [23]. The endothelial differentiation of CSCs was then further supported with the use of a genetic mouse model [24]. The authors successfully traced tumor initiating cells in the brain as the cell origin of endothelial cells in GBM and excluded the possibility of cell fusion. This transdifferentiation is enhanced by the induction of HIF-1α, and it is VEGF-independent [24]. These studies demonstrate that CSCs in multiple tumors have endothelial differentiation abilities and directly contribute to tumor angiogenesis. Furthermore, these results also partially explain why anti-VEGF therapy is not very efficient in clinical application because the endothelial differentiation of CSCs is VEGF-independent.

### Pericytes

Pericytes attach to endothelial cells to support the neovasculature, maintain the integrity of blood vessels and communicate with each other to regulate endothelial homeostasis [51, 52]. A low number of vessel-associated pericytes or the absence of pericyte coverage is correlated with increased metastasis in colorectal, prostate, pancreatic and breast cancers [53]. The current study suggests that pericytes originate from pericyte progenitors in surrounding normal tissues or from bone-marrow-derived cells (BMDCs) in tumors. A previous report demonstrated that neural stem cells have the ability to transdifferentiate into pericytes, indicating that normal somatic stem cells are capable of generating mural cells [54]. Therefore, it is possible that CSCs also have the potential to transdifferentiate into mural cells. GSCs were firstly reported to transdifferentiate into mural cells in 2012 [55]. GSCs gain mural cell markers, but not endothelial cell markers, upon transdifferentiation which is regulated by Flk-1. Despite its novelty, several questions still remained unsolved in the referenced report. The authors did not identify the specific type of mural cells that resulted from transdifferentiation; were they vascular smooth muscle cells or pericytes? The authors did not determine whether this transdifferentiation is independent of cell fusion, and they did not provide evidence supporting whether mural cells in clinical samples carry the same genetic mutations as the tumor cells. Bao and colleagues first demonstrated that GSCs can transdifferentiate specifically into pericytes [25]. GSCs can give rise to a pericyte lineage *in vitro* and in xenograft model *in vivo*. This work also took advantage of a lineage-specific fluorescence reporter system to validate that GSCs have the capacity to transdifferentiate into pericytes *in vivo*. By analyzing patients' GBM samples, the authors found that vast majority of tumor pericytes carry the same genetic alternations as neoplastic cells. Moreover, selectively targeting GSC-derived pericytes disrupts tumor vessels and inhibits tumor growth. Interestingly, GSCs preferentially transdifferentiate into pericytes but not into endothelial cells. Although cells expressing endothelial markers were detected in the cells that were differentiated from GSCs in culture, the numbers were very low [25]. The authors further found that GSCs express CXCR4 whereas endothelial cells secret SDF-1. Thus, GSCs are recruited towards endothelial cells through the SDF-1/CXCR4 axis. With the stimulation of TGF-β, GSCs give rise to pericytes, indicating that transdifferentiation of GSCs can be regulated by cell-intrinsic or microenvironmental cues [25]. However, it remains unknown whether transdifferentiation of CSCs into pericytes is present in other types of tumors.

### Cancer-associated fibroblasts

CAFs are a major type of stromal cells in the tumor microenvironment [56]. There are several potentially reliable sources of CAFs. Resident normal fibroblasts are believed to be the major source of CAFs in tumors because CAFs share several similarities with normal fibroblasts [57]. Some mesenchyme cells are also thought to be one of the sources of CAFs. Smooth muscle cells are hypothesized to covert to CAFs in prostate cancer stroma [58]. Myofibroblasts are documented to acquire a CAF phenotype in the presence of appropriate stimuli [59]. Fibrocytes are also reported to contribute to CAFs in invasive ductal carcinoma of breast [60]. Pericytes [61] and adipose tissue-derived stem cells [62] can transdifferentiate into CAFs, although this transdifferentiation requires further experimental support. Moreover, CAFs can be recruited from distant organs and derived from BMDCs and MSCs [62, 63]. In addition, the endothelial-to-mesenchymal transition also generates CAFs, which is validated in mouse models [64]. This discovery makes endothelial cells a reliable source for CAFs. Interestingly, epithelial cells in the tumor may also be a source of CAFs. Epithelial cells in carcinoma alter their differentiation status especially through undergoing epithelial-mesenchymal transition (EMT). Although EMT is difficult to trace *in vivo*, numerous *in vitro* studies demonstrate that EMT is a well-defined program in carcinoma. By the EMT program, epithelial cells exhibit fibroblast-like morphology and express mesenchymal markers, such as α-SMA, and acquire partial stem cell-like properties. In breast cancer, EMT provides a nonmalignant stroma. The α-SMA-expressing cells that have originated from epithelial tumor cells promote tumor growth in nude mice [65]. Another study also supports that epithelial cells may be one source of CAFs. Twenty-four percent of CAFs are GFP-labeled cancer cells [66]. Although EMT-transformed cancer cells can serve as CAFs, there is no direct evidence that CSCs transdifferentiate into CAFs *in vivo*. Recent studies reveal that constitutively activating EMT suppresses the stemness of human epithelial CSCs *in vitro* and inhibits their metastatic colonization abilities *in vivo*; however, transiently inducing EMT at primary site enhances local invasion and blood entry [67, 68]. Tsuji *et al*., showed that only a mixture of EMT and non-EMT HCPC-1 cells can form lung metastases in nude mice and that neither EMT nor non-EMT HCPC-1 cells establish lung metastases when injected separately [69]. In addition, EMT-derived CAFs have multiple genetic mutations that are similar to cancer cells, while other sources of CAFs need to acquire extra genetic and/or epigenetic hits to undergo malignant transformation. As a result, EMT may be a transdifferentiation program for CSCs and/or progenitors to generate CAFs in some tissue contexts to promote tumor growth and metastasis instead of only recruiting fibroblasts from local or distant tissues during tumor progression. Further study is needed to determine whether these CAFs originate from CSCs and the functions of these CSC-derived CAFs in tumor progression.

#### MSCs and MSC-derived cells in tumor

Accumulating evidence suggests that MSCs or MSC-derived cells play important roles in tumor growth and metastasis. MSCs are present in various organs in human adults; however, the major cell origin of MSCs in the tumor microenvironment is bone marrow. Bone marrow-derived MSCs have different cell fates in the tumor microenvironment in which they can differentiate or transdifferentiate into CAFs, endothelial-like cells, pericytes and macrophage-like cells [70]. Bone marrow-derived MSCs are recruited to the tumor microenvironment in different solid tumors by multiple chemokines, cytokines and growth factors. When recruited to the tumor sites, MSCs and their derived cells interact with tumor cells and other types of cells in the tumor microenvironment. At primary sites, MSCs can induce EMT of cancer cells in hepatocellular carcinoma and breast cancer as well as promote the metastatic ability of colon and ovarian cancer cells [62]. When tumor cells evade from primary sites, MSCs construct an immunosuppressive microenvironment by inhibiting the function of immune cells and helping tumor cells escape from immune surveillance. Moreover, MSCs are also involved in the formation of metastatic niches. MCSs facilitate the colonization of CSCs and provide a niche for CSC self-renewal, promoting tumor initiation and metastasis of colon and breast cancers [71].

Because CSCs have multi-lineage differentiation abilities, CSCs might be one of the sources of MSCs and MSC-derived cells. GSCs have mesenchymal-lineage differentiation abilities in orthotopic *versus* heterotopic xenograft mouse models and *in vitro* differentiation analyses [72]. Subcutaneous injection of CSCs or single CSC clones can produce tumors with osteochondrogenic areas, which further confirms this observation. Mani and colleagues have reported that EMT-derived human mammary epithelial cells express several surface markers that are similar to MSCs, and they differentiate into adipocytes, osteocytes and chondrocytes *in vitro* as well as contribute to wound healing *in vivo* [73]. Our data reveal that human mammary epithelial cells and breast cancer cells are induced to gain some multi-lineage differentiation capabilities of MSCs *in vitro* [74]. Small lung cancer cells NCI-H446 can transdifferentiate into osteocytes and adipocytes *in vitro* [75]. PC-3 and DU-145 prostate cancer cells can undergo osteoblastogenic and adipogenic differentiation, while LNCaP prostate cancer cells only give rise to osteoblasts [76]. Individual melanoma cells from melanoma spheres also undergo adipogenic, osteogenic and chondrogenic differentiation [77]. These observations that cancer cells or CSCs in various cancers display the multi-lineage differentiation potentials similar to MSCs indicate that CSCs may serve as MSC-like cells in the tumor microenvironment to promote tumor growth and metastasis. Although CSCs exhibit partial MSC properties and give rise to MSC-derived cells, it remains elusive whether these CSC-derived cells function similarly to MSCs or MSC-derived cells. A recent report showed that MSCs fuse with breast cancer cells, making breast cancer cells gain partial mesenchymal phenotypes [78]. Therefore, further work is needed to determine whether CSCs generate MSC-like cells in the tumor microenvironment.

#### Neural lineages

Nerves are a common component of the tissue microenvironment and they play important roles in regulating cell behaviors and tissue homeostasis; however, their roles in tumorigenesis and metastasis are poorly understood. Different groups have reported that the nervous system contributes to tumor growth and metastasis [79]. Autonomic nerve development in prostate cancers has been demonstrated to promote tumor growth *in situ* as well as facilitate tumor invasion and dissemination [80]. Embryonic stem cells and various adult stem cells can differentiate into neurons, astrocytes and oligodendrocyte lineages *in vitro* and *in vivo*; however, whether CSCs in non-brain tumors can transdifferentiate into neurons or other neural lineages remains unclear. Some cancer cells display components of the phenotypes of neurons; for example, breast-to-brain metastatic cells exhibit GABAergic characteristics that are similar to neuronal cells to promote their metastatic growth [81], indicating that CSCs may gain some phenotypes of neural cells in the tumor microenvironment. Prostate CSCs can differentiate into luminal secretory epithelial cells and transdifferentiate into terminal differentiated neuroendocrine cells [82]. Several reports have demonstrated neuron differentiation ability of CSCs *in vitro*. WER-RB1 CSCs differentiate into neuron-like cells in neuronal-medium and express GFAP *in vitro* [83]. In addition, treating NCI-H466 with trichostatin A *in vitro* induces a neuron-like phenotype as well as the expression of multiple neural cell markers BM88 and NF-200 [75]. Ovarian CSC-derived CP70SR01 clones also exhibit neuron-like morphology under modified induction medium and express α-internexin and pan-neuronal markers *in vitro* [84]. Because tumor growth and metastasis need stimulations derived from neurons, it is possible that cancer cells, in some contexts, gain access to sufficient stimulation by directly transdifferentiating into neuron-like cells from CSCs during tumor growth and metastasis. Although the neuron differentiation of CSCs has been detected in several types of cancer cells *in vitro*, there is still insufficient direct evidence to demonstrate that CSCs generate functional neuron-like cells in the tumor microenvironment *in vivo*.

#### Immune inflammatory cells

The tumor microenvironment contains innate immune cells (such as macrophages, myeloid-derived suppressor cells (MDSCs) and dendritic cells) and adaptive immune cells (T and B cells) [85, 86]. The major immune cell types in the tumor microenvironment are macrophages, MDSCs and T cells. Tumor-associated macrophages (TAMs) play important roles in tumor growth and metastasis [87]. MDSCs, newly discovered myeloid cells in the tumor microenvironment, can promote angiogenesis and metastasis as well as help cancer cells escape from immune surveillance by suppressing the anti-tumor activity of natural killer cells and cytotoxic T cells [88]. T cells have tumor-promoting and tumor-antagonizing activities [86]. TAMs are believed to originate from hematopoietic stem cell-derived monocytic and granulocytic progenitors in bone marrow or monocytic and granulocytic progenitors in spleen [89]. As for MDSCs, hematopoietic stem cells give rise to common myeloid cells and then differentiate into immature myeloid cells. These immature myeloid cells are recruited into tumor microenvironment by different cytokines and chemokines and are maintained as MDSCs [90]. Multipotent lymphoid progenitors are the cellular origin of T lymphocytes. These lymphoid progenitors migrate from the bone marrow to thymus and undergo differentiation into pre-T cells. Pre-T cells then undergo positive or negative selection to generate mature T cells [91]. Immune cells in the microenvironment generally originate from hematopoietic stem cells in the bone marrow. Although the transdifferentiation of CSCs into macrophages, MDSCs or T cells has not been reported thus far, several interesting pieces of evidence support this possibility. First, cell fusion of cancer cells, epithelial cells or stem cells with BMDCs, especially macrophages, is a common event in tumorigenesis and metastasis [92]. Fusion between epithelial cancer cells and macrophages was reported in the intestine, which generates hybrid cells that have the characteristics of cancer cells and macrophages upon nuclear reprogramming [93]. These hybrid cells obtain higher metastatic abilities and immune evasion capacities. Fusion between cancer cells and CD45^+^ hematopoietic cells has also been observed in ovarian carcinoma. These cell fusion-derived epithelial cancer cells co-express epithelial and hematopoietic cell markers and exhibit elevated stemness and migratory abilities [94]. Cell fusion between cancer cells and immune cells generates hybrid cells with the properties of parental cells. We speculate that CSCs may fuse with immune cells or directly transdifferentiate into immune cells to gain the characteristics of immune cells, instead of only recruiting immune cells from distant bone marrow, in some cancer contexts. The local tumor microenvironment contains various cytokines and chemokines that may induce this transdifferentiation. As CSCs have multi-potent differentiation abilities and high metastatic capabilities, this potential transdifferentiation seems to be more a realistic and economical choice at certain stages of tumor initiation or progression. Interestingly, BMDCs have been discovered to be the cell origin of gastric cancer. Under *Helicobacter* infection, which induces chronic inflammation, BMDCs home to the stomach and initiate tumorigenesis through metaplasia and dysplasia to intraepithelial cancer in a cell fusion-dependent manner [95]. In this tumor model, BMDCs exhibit similarities, such as long-term self-renewal, maintenance of the undifferentiated state, transdifferentiation and metastasis-prone abilities. This observation raises the possibilities that CSCs may have the potential to give rise to BMDCs in the tumor microenvironment because there is commonality between CSCs and BMDCs. It is also of interest to investigate whether the conversion between CSCs and BMDCs is present in the tumor microenvironment. In addition, macrophages and MDSCs are involved in the regulation of CSC niches. TAMs promote self-renewal and the antidrug resistance of CSCs through paracrine signaling in the murine colon and breast carcinoma [96, 97]. MDSCs enhance the stemness of ovarian CSCs by inducing the expression of miR-101 [98]. As a result, CSCs probably transdifferentiate into TAMs or MDSCs to interact with CSC niches and maintain the CSC pool. Due to the lack of direct evidence, further study is needed to illustrate whether CSCs give rise to BMDCs and other tumor-associated immune cells *in vitro* and *in vivo*.

## EXPERIMENTAL REPROGRAMMING OF CANCER CELLS

Cancer can be regarded as a disease of reprogramming and differentiation [99, 100]. Differentiated cancer cells can dedifferentiate into CSCs or CSC-like cells through transcriptional regulation, post-transcriptional regulation, microenvironment signal stimulation, epigenetic modification and metabolic reprogramming [100]. Subpopulations of renal tumor cells, medulloblastoma cells and RAS-induced melanoma cells have been successfully reprogrammed to pluripotency by somatic nuclear transfer [101,102]. Because mouse and human somatic cells can be reprogrammed to induced pluripotent stem (iPS) cells by Oct4, Sox2, Klf4 and C-myc [103], it is reasonable to speculate that cancer cells can be reprogrammed to pluripotent state through inducing expression of Yamanaka factors. Indeed, chronic myeloid leukemia (CML)-iPS cells has been generated from blast crisis stage CML cells by retrovirus infection of Yamanaka factors. These CML-iPS cells give rise to teratomas in NOD-SCID mice and differentiate into hematopoietic lineages *in vitro* [104]. Miyoshi et al., reprogrammed gastrointestinal cancer cells into iPS cells and these iPS cells generate three germ layer cells *in vitro* [105]. GBM neural stem cells [106] and sarcoma cells [107] have also been reprogrammed to iPS cells. Kim et al., introduced expression of Yamanaka factors in primary pancreatic epithelial cells isolated from human pancreatic ductal adenocarcinoma (PDAC) and successfully generated a patient-derived iPS cell line [108]. This patient-derived cancer iPS cell line can differentiate into three germ layer cell linages *in vitro* and generate multiple germ layer tissues *in vivo*. Moreover, endodermal ductal structure generated by this iPS cell line *in vivo* develops into pancreatic intraepithelial neoplasia in 3 months and progresses into invasive carcinoma by 9 months, indicating that cancer iPS cells mimic tumor initiation and progression *in vivo* [108]. In addition, the EMT program generates CSCs from cancer cells [16]. Therefore, experimental reprogramming of cancer cells into iPS cells or CSCs will provide new insights into tumor initiation, early progression and metastasis and help us to further demonstrate that cancer is a disease of dysregulated reprogramming and differentiation.

## FUTURE PERSPECTIVES

Tumors can be considered as a complex organ that contains heterogeneous cancer cells and other types of stromal cells; these cells, together with the ECM and local and systemic factors, construct the tumor microenvironment. It is well-defined that cancer cells recruit multiple types of stromal cells from local tissues or bone marrow to construct their own microenvironment [109]. The genomic instability and differences in the microenvironment contribute to the phenotypic and functional heterogeneity of cancer cells [110]. Understanding the interactions between cancer cells and tumor stromal cells in the tumor microenvironment as well as their functions in tumor growth and metastasis are critical for unraveling the complexity of tumors and developing novel strategies for anti-cancer therapy. The emerging CSC concept provides new insight into the theory on tumor heterogeneity and tumor microenvironment. Although the CSC model may be not appropriate for all tumor types, emerging evidence supports the presence of CSCs in a variety type of tumors. The CSC theory also elucidates many unsolved problems in basic and clinical cancer research, such as the cellular origin of tumors, tumor heterogeneity, drug resistance, tumor metastasis and relapse. However, the genetic heterogeneity of CSCs makes it difficult to identify CSCs in tumors because of the lacking of solid biomarkers [111].

**Figure 3:**
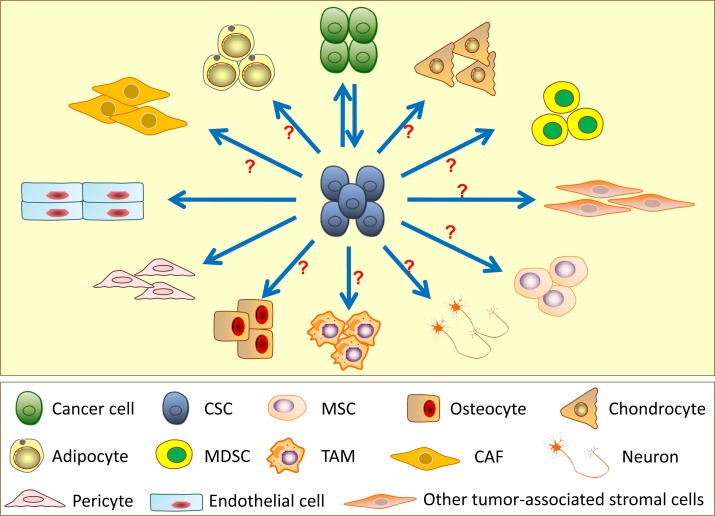
Differentiation and transdifferentiation potentials of cancer stem cells (CSCs) CSCs can differentiate into the non-stem original cell lineages from which the tumor arose as well as have potential to transdifferentiate into other cell lineages, such as endothelial cells, pericytes, cancer-associated fibroblasts (CAFs), mesenchymal stem cells (MSCs), adipocytes, osteocytes, chondrocytes, myeloid-derived suppressor cells (MDSCs), tumor-associated macrophages (TAMs) and neurons in specific tissue contexts. However, there is insufficient direct evidence supporting the transdifferentiation of CSCs into most of other cell lineages.

Current evidence supports that CSCs can regenerate cancer cells *in vitro* and *in vivo*, which strictly follows the CSC model. Emerging data also provide evidence that CSCs have the potential to transdifferentiate into numerous types of stromal cells in the tumor microenvironment even though most of the lineage transdifferentiation from CSCs is still unproven (Figure 3). It has been demonstrated that CSCs give rise to endothelial cells *in vitro* and *in vivo*. This transdifferentiation is observed in GBM, breast, ovarian and renal cancers, although the transdifferentiation efficiency is believed to be relatively low. Moreover, CSCs can generate pericytes in GBM. These findings update the basic principle of the cell origin in tumor angiogenesis. In addition, CSCs have been demonstrated to give rise to mesenchymal lineage cells, such as adipocytes, osteoblasts and osteoclasts, *in vitro*. Several pieces of evidence indicate that adipocytes and neurons promote tumor growth, metastasis and drug resistance in different tumors [80, 112]. Osteoblasts and osteoclasts are involved in the bone metastasis of CSCs. Therefore, there might be a complex relationship and crosstalk between CSCs and these stromal cells. In addition, CSCs may have the potential to transdifferentiate into CAFs, TAMs, BMDCs and other cells; however, currently, we lack direct evidence to confirm the transdifferentiation.

Although the transdifferentiation of CSCs into tumor stromal cells is compelling and provides a new dimension explaining tumor heterogeneity, many aspects of CSC transdifferentiation remain elusive. First, CSC transdifferentiation needs to be further elucidated *in vivo* using genetic mouse models and clinical samples. Taking advantage of lineage-tracing approaches in mice will help us gain a better understanding of cell fates of CSCs. We also should determine the transdifferentiation efficiency of different cell types in different tumors. Second, understanding the molecular mechanism underlying CSC transdifferentiation is also important for deciphering tumor heterogeneity and developing new therapeutic targets. Currently, we are still confused about when and how CSCs undergo multi-lineage differentiation in the tumor microenvironment to promote tumor growth and progression. It is apparent that CSC transdifferentiation is regulated at the genetic and epigenetic levels. It is also expected that CSC transdifferentiation is tightly controlled temporo-spatially. It is urgent to determine the types of regulators and molecular switches that are responsible for this transdifferentiation scenario. These observations also lead to another thought about whether these transdifferentiation-derived cells can dedifferentiate into CSCs or transdifferentiate into other types of cells. If this is true, we will find that multi-sources of cells in the tumor microenvironment contribute to the complexity of tumor heterogeneity.

CSC transdifferentiation may have tremendous clinical implications in cancer treatment. Current strategies for anti-cancer therapy mainly focus on eradicating the fast-dividing cells or differentiated cells; however, these therapeutics have minimal effect on those CSCs that are slowly dividing or that remain quiescent. Increasing evidence suggests that CSCs are significantly insensitive to multiple therapeutics [113, 114]. Moreover, chemotherapy may enrich the CSC pool, which is responsible for tumor recurrence after combined anti-cancer therapy [12, 115]. Therefore, selectively targeting CSCs may be a promising strategy to cure cancer. However, targeting CSCs remains difficult [114, 116]. In addition, there is evidence shows that cancer stem-like cells (stemloids) are different from CSCs which are usually quiescent. Cancer stem-like cells are a group of proliferating self-renewing cells expressing cancer stem cell markers. Stemloid-based relapse is more aggressive than CSC-based relapse [117,118]. CSCs reside in CSC niches and the CSC niches are critical for CSCs to maintain stemness [119]. Non-CSCs in the tumor microenvironment can protect CSCs from chemotherapy [120]. Because CSCs have the potential to transdifferentiate into stromal cells in tumor microenvironment, selectively targeting these CSC-derived cells may disrupt the CSC niches. Therefore, simultaneously targeting CSCs and non-CSCs may offer better strategy for cancer therapy. However, we still lack target drugs that are specific for CSCs. Therefore, inducing the differentiation of CSCs and targeting the CSC-derived stromal cells may have a clinical benefit for treating cancer. This prompts us to ask about the functional differences between the CSC-derived stromal cells and their “normal” stromal counterparts. Further understanding the molecular signature will help us to develop better therapeutics against CSC-derived stromal cells and CSCs themselves.

In conclusion, the observations of the transdifferentiation of CSCs into multiple stromal cells in tumors suggest that the plasticity and functional heterogeneity in cancer cells and their stromal compartments is far more complex than we expected. Although this CSC transdifferentiation still remains elusive in many aspects, the discoveries in this field will advance our understanding of the plasticity and heterogeneity of tumors and may help with developing new diagnostic and therapeutic strategies against tumors.
